# A comparison of cemented and cementless intra-neck curved stem use during hip-preserving reconstruction following massive femoral malignant tumor removal

**DOI:** 10.3389/fonc.2022.933057

**Published:** 2022-09-05

**Authors:** Qi You, Minxun Lu, Li Min, Yi Luo, Yuqi Zhang, Yitian Wang, Chuanxi Zheng, Yong Zhou, Chongqi Tu

**Affiliations:** ^1^ Department of Orthopedics, Orthopedic Research Institute, West China Hospital, Sichuan University, Chengdu, China; ^2^ Sichuan Model worker and Craftsman Talent Innovation Research Studio, Chengdu, China

**Keywords:** massive femoral malignant tumor, total femur replacement, customized femoral endoprosthesis, intra-neck curved stem, hip-preserving reconstruction

## Abstract

**Background:**

Patients who undergo massive femoral malignant tumor (MFMT) resection often exhibit shortened femoral metaphyseal juxta-articular segments. The use of a customized femoral endoprosthesis (CFE) with an intra-neck curved stem (INCS) has emerged as a viable reconstructive surgical strategy for these individuals. Relative to a cemented INCS, it remains unclear as to whether cementless INCS use is associated with improvements in functionality or reconstructive longevity. As such, the present study was conducted to compare functional outcomes, endoprosthetic survival, and endoprosthesis-related complication rates in patients undergoing cemented and cementless INCS implantation.

**Methods:**

A total of 24 patients undergoing lower limb salvage and reconstructive surgical procedures utilizing cemented or cementless INCS endoprostheses were retrospectively included. Patient-functional outcomes, endoprosthetic survival, and complication rates were compared as a function of age; diagnosis; the length of femoral resection; residual proximal femur length; Musculoskeletal Tumor Society (MSTS) scores; visual analog scale (VAS) scores; and the rates of implant breakage, periprosthetic infections, periprosthetic fractures, and aseptic loosening.

**Results:**

The mean follow-up was 56 months. Significant differences in the length of femoral resection (p<0.001) and residual proximal femur length were observed (p<0.001) between the cemented and cementless INCS groups. There were no differences in overall patient survival and aseptic loosening-associated endoprosthesis survival in the cemented and cementless groups. None of the included patients experienced periprosthetic fractures, infections, or implant breakage. Average respective MSTS and VAS scores did not differ between groups.

**Conclusion:**

For patients undergoing treatment for MFMTs, the use of a CFE with an INCS has emerged as a viable approach to hip-preserving reconstructive surgery. With appropriately designed individualized rehabilitative programs, good functional outcomes can be achieved for these endoprostheses, which are associated with low complication rates. Moreover, the selection between cemented or cementless INCS in the clinic should be made based on patient-specific factors, with cementless INCS implementation being preferable in younger patients with good-quality bone, the potential for long-term survival, and the osteotomy site near the lesser trochanter, whereas cemented INCS use should be favored for individuals who are older, have a shorter life expectancy, or have poor bone quality.

## Introduction

In recent years, improvements in tumor staging, adjuvant treatment options, and endoprosthetic design have made limb salvage surgery an increasingly viable option for patients undergoing treatment for massive femoral malignant tumors (MFMTs) (1). Conventionally, total femur replacement (TFR) has been the primary approach to limb salvage in these patients, but this strategy is associated with complications including local recurrence, infection, aseptic loosening, hip disarticulation, and the potential for limb-length discrepancies (2–4). Proximal femoral resection also often necessitates the opening of additional tissue compartments, potentially leading to the hip joint being affected in cases of infection or disease recurrence, ultimately resulting in the need for a hemipelvectomy to achieve the margins necessary to avoid further local recurrence (5). The proximal femur is subject to high levels of biomechanical stress and is the point of contact for several soft tissues necessary for appropriate lower limb function (6). The preservation of native hip joints can lower the risk of muscle damage, surgical disruption, and articular surface degeneration relative to the use of endoprosthetic hip joints, thereby improving the overall function of the lower extremities in treated patients. Alternative approaches to hip-preserving reconstruction (HPR) following MFMT resection include the use of inactivated autologous bone grafts (7), osteoarticular allografts (8), combined autografts and allofraft (9, 10), and endoprostheses. Inactivated autologous bone grafted has the advantage of anatomical matching, biological reconstruction, relatively low cost, and no need for large bone bank support; however, inactivated bone fracture, infection, non-union, and internal fixation failure are the disadvantages of this technique (7). Osteoarticular allograft can result in the biological reconstruction of bone defects and preserve host bone stock without donor site morbidity (11). Massive allografts are associated with a high risk of infection, graft fracture, and delayed union or non-union (8, 12). Combined autografts and allografts combine the biological activity of free vascularized fibular grafts (FVFGs) with the initial mechanical strength of allografts. The Capanna technique has been reported to lessen the impact of complication (graft fracture and delayed union or non-union) (9, 13–15). However, the risk of anastomosis failure by thrombosis is a concern (16). In addition, a previous study showed that there was little difference in the percentage of graft fractures when comparing allografts with and without this vascularized graft (17).

While endoprosthetic reconstructive surgery is associated with excellent cosmetic, functional, reliability, and emotional acceptance outcomes in the short term (18), it can also be subject to long-term complications including infection, endoprosthetic joint dislocation, aseptic loosening, and mechanical breakage, all of which can contribute to implant failure (1). Cemented fixation strategies have remained the gold standard approach since the advent of limb salvage surgery, enabling rapid weight-bearing and stability without restriction while effectively adapting to the bones of varying geometry and quality characteristics. It also allows for drug delivery and can be less costly than cementless fixation procedures (19). Even so, late aseptic loosening is a relatively common complication in patients who undergo cemented tumor endoprosthesis implantation (1). As cementless fixation strategies allow for the ingrowth of new bone, they may be advantageous, contributing to lower aseptic loosening rates (19, 20). In our institution, we defined a short proximal femur (SPF) as the length of the residual proximal femur of ≤110 mm (the length from the pyriform fossa to the osteotomy level). The residual SPF segment may be insufficient to accept a standard 150-mm intramedullary cemented stem (21). To make the endoprosthetic stem better match the curvature of the residual proximal femur and increase the contact area between the endoprosthetic stem and cancellous bone, we utilized a customized femoral endoprosthesis with an intra-neck curved stem (INCS) to reconstruct massive femoral diaphyseal defects with an SPF. To our knowledge, no studies to date have specifically compared functional outcomes, endoprosthetic survival, and endoprosthesis-related complication rates in MFMT patients undergoing HPR using cemented or cementless INCS-based reconstructive approaches.

The present study was developed to explore both oncological and functional outcomes in MFMT patients undergoing HPR using cemented or cementless INCS, with a specific focus on endoprosthetic survival, patient-functional outcomes, and postoperative endoprosthesis-related complications through a direct comparison of these two patient groups.

## Materials and methods

### Patients

**Figure 1:**
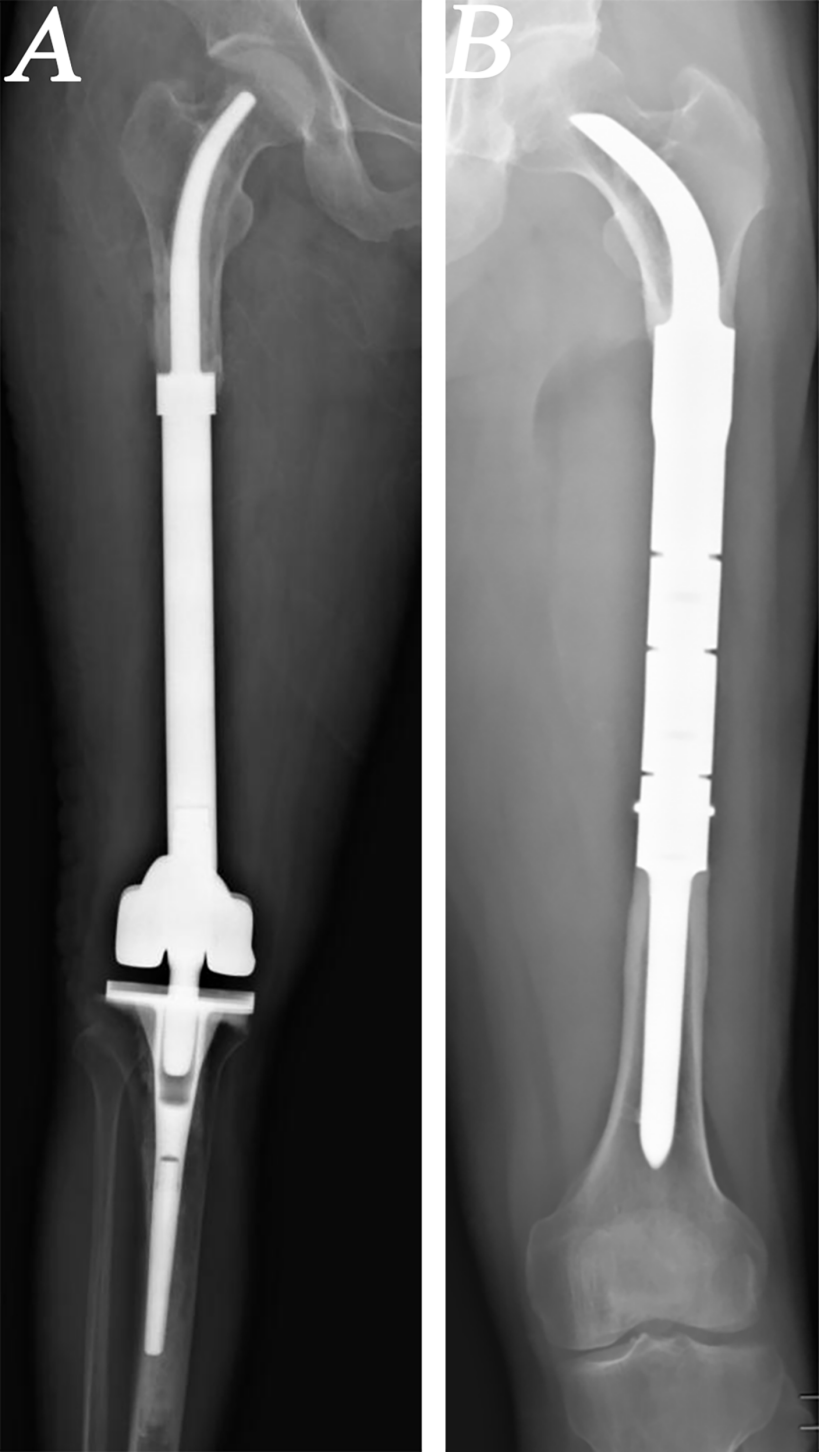
Radiographs of patients who underwent hip-preserving reconstruction (HPR) with intra-neck curved stem (INCS). **(A)** A patient who underwent HPR with cemented INCS. **(B)** A patient who underwent HPR with cementless INCS. (HPR: hip-preserving reconstruction; INCS: intra-neck curved stem).

From 2013 to 2019, a total of 24 patients with MFMTs underwent HPR using a customized femoral endoprosthesis (CFE) with a cemented or cementless INCS at the author’s institution (**Figure 1**). These patients (14 males, 10 females) had a mean age of 24.5 years (range: 10–62 years) and an average follow-up duration of 56 months (range: 17–102 months). Surgical staging was performed with the Enneking bone and soft tissue sarcoma staging system (22). All patients underwent biopsy prior to definitive surgery, with preoperative X-ray, computed tomography (CT), single-photon emission CT, and magnetic resonance imaging (MRI) approaches being used to measure the length of bone to be resected. Patient demographic characteristics (age, sex), defect length, and residual proximal femur length were recorded (**Table 1**).

**Table 1:** Patient characteristics of study population.

	N (%)			
Characteristic	Total (N=24)	HPR with cemented INCS (N=11)	HPR with cementless INCS (N=13)	*p*
Age, mean (SD) (y) Diagnosis Osteosarcoma Ewing sarcoma *Other Stage I IIA IIB III Length of femur resection, mean (SD) (mm) Length of residual proximal femur, mean (SD) (mm)	24.5 (13.3) 12 (50) 7 (29) 5 (21) 0 0 22 (92) 2 (8) 240.2 (102.4) 79.3 (14.5)	25.7 (14.7) 6 (55) 3 (27) 2 (18) 0 0 10 (91) 1 (9) 337.4 (16.3) 90.6 (10.6)	23.5 (12.6) 6 (46) 4 (31) 3 (23) 0 0 12 (92) 1 (8) 158.0 (62.2) 69.7 (9.5)	0.698 0.915 0.902 <0.001 <0.001

*Other primary bone sarcoma included either “high grade, undifferentiated sarcoma” or “high grade spindle cell neoplasm” based on available pathology reports. HPR: hip-preserving reconstruction; INCS: intra-neck curved stem; SD: standard deviation.

The ethics committee of the author’s institution approved this retrospective study, with all patients having provided written informed consent.

### Stem design and fabrication

For patients undergoing implantation procedures using a cemented INCS, stems were designed as arc-shaped solid structures with a diameter 2–3 mm less than that of the inner surface of the inner femoral cortices. The curvature of the developed stem was based upon the medial cortex of the femoral neck. To maintain strength, the stem diameter was gradually reduced such that there was a >10-mm diameter remaining at the end of the curved portion of the stem. Arc-shaped stems were used for patients undergoing implantation procedures using a cementless INCS, with the center of the stem being solid, with the medial and lateral stem porosity values of 50% and 70%, respectively. The base of the curved stem was designed with two fins, and the diameter at the stem base was identical to that of the inner surfaces of the femoral cortices. Stem strength was maintained by gradually reducing the curved stem diameter between the intertrochanteric region and femoral head–neck junction, with a stem diameter at the latter point that was roughly two-thirds the diameter of the medullary cavity. Stem curvature was primarily based upon the shape of the femoral neck medial cortex, and the diameter at the end of the curved stem remained >10 mm. The tip of this curved step exhibited a beak-like shape. All stems were designed by our clinical team and fabricated by Chunlizhengda Medical Instruments (Tong Zhou, Beijing, China).

### Surgical approach

The same senior surgeon performed all procedures included in this study. Patients were placed in a lateral recumbent position, and a lateral approach was used for all patients. En bloc tumor resection and the removal of soft tissue were performed based upon preoperative simulation results. The potential for endoprosthetic misfit was minimized by carefully controlling the degree of the osteotomy plain. Following tumor resection, patients undergoing cemented INCS implantation had the tip of a customized guide needle inserted into the center of the femoral head with a mobile C-arm, with the medullary cavity being gradually enlarged using a customized guide needle and flexible reamers of different diameters to generate a cone-shaped cavity. The prosthesis was then prepared to match the residual femoral curvature and to appropriately correct for the alignment of the lower extremities. Bone cement was then injected into the medullary cavity with a vacuum-mixing gun, and the curved stem was then inserted into the residual proximal femur, ensuring that no cement remained present between the endoprosthesis and soft tissues. For patients undergoing cementless INCS implantation, a bone curette was used to remove cancellous bone from the osteotomy surface, after which a mobile C-arm was used to insert a customized guide needle into the center of the femoral head. Flexible reamers and customized guide needles were then used to generate a cone-shaped medullary cavity as above. To ensure maximal endoprosthetic stability while minimizing bone loss, the residual proximal femur was under-reamed by 0.5 mm. Additional reaming was then conducted at 0.5-mm increments as necessary to achieve a stable fit. To initially verify matching between the INCS and the proximal femur, a smaller plastic trial-model endoprosthesis was initially inserted. Appropriately sized endoprostheses were then implanted, with harvested cancellous bone being used for grafting.

### Postoperative management

Patients were routinely administered prophylactic antibiotics for 48 h postsurgery. Individualized assessments were used to guide the development of patient-specific rehabilitative programs. For patients who have undergone procedures using a cemented INCS, patients were confined to bed rest for 3–5 days with their lower extremities in a neutral position. After 8 h, these patients began ankle and knee flexion and extension exercises, progressing to hip abduction and flexion exercises after 3 days, and initiating partial weight-bearing after 7 days using two crutches for assistance. Progression to full weight-bearing was initiated after 21 days. For patients that have undergone procedures using a cementless INCS, patients were confined to bed rest for 2–3 weeks with their lower extremities in a neutral position. During this recuperative period, ankle and knee flexion and extension exercises were initiated. After 3 weeks, partial weight-bearing using crutches was initiated, while hip abduction and flexion exercises were initiated after 4 weeks. After 8 weeks, patients progressed to walking with partial weight-bearing using one crutch, further progressing to full weight-bearing after 12 weeks.

Patients underwent monthly follow-up for 3 months postsurgery, after which they underwent a follow-up evaluation every 3 months for 2 years, followed by annual follow-up visits thereafter. The affected limb was evaluated during each follow-up, with pain being rated using a VAS. Radiographic imaging of the reconstructed limb was performed monthly for the first 3 months, every 3 months for the remainder of the first year, every 6 months during the second year, and annually thereafter. Lower limb function was assessed using the MSTS scoring system (23). Patients were monitored for INCS implantation–associated complications such as infection, implant breakage, aseptic loosening, and periprosthetic fracture.

### Statistical analysis

Data were compared *via* two-sample t-tests, chi-square tests, or the Fisher’s exact test, with p < 0.05 as the significance threshold. SPSS v 19.0 (IBM Corp., Armonk, NY, USA) was utilized for all data analyses.

## Results

A total of 11 and 13 patients in the present study cohort underwent HPR using a CFE with a cemented and a cementless INCS, respectively. None of these patients exhibited preoperative pathologic fractures. The mean respective follow-up durations in the cemented and cementless INCS groups were 63.5 and 49.7 months (p=0.175). The mean ages of patients in these two groups were 25.7 and 23.5 years, respectively (P=0.698). Patients in the cemented INCS and cementless INCS groups exhibited respective mean femoral resection length values of 337.4 mm and 158.0 mm, respectively (p<0.001), with corresponding mean residual proximal femur length values of 90.6 and 69.7 mm, respectively (p<0.001).

Average respective MSTS scores in the cemented INCS and cementless INCS groups were 24.9 and 26.0 (p=0.168), and none of the surviving patients in either group required the use of crutches or other walking aids as of most recent follow-up. Two patients in the cemented INCS group reported mild pain in the lower extremities when walking unsupported for >3,000 m, with VAS scores of 3 and 2, respectively. These patients ultimately underwent revision surgery due to aseptic loosening. One patient in the cementless INCS group reported mild pain in the lower extremities when walking unsupported for >5,000 m (VAS score = 2 at final follow-up) but did not exhibit any imaging complications associated with endoprosthesis implantation. No other patients reported pain or Trendelenburg gait as of most recent follow-up.

**Figure 2:**
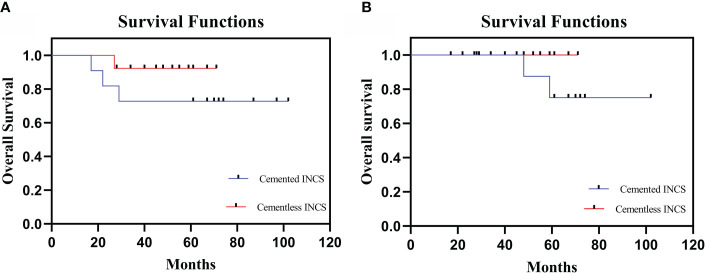
**(A)** The overall patient survival of HPR with cemented INCS versus cementless INCS. **(B)** Survival to aseptic loosening of cemented INCS versus cementless INCS. (HPR, hip-preserving reconstruction; INCS, intra-neck curved stem).

Three patients in the cemented INCS group have died of lung metastases at 17, 22, and 29 months postreconstruction, while one patient in the cementless INCS group died of lung metastases at 27 months postreconstruction (p=0.300). Aseptic loosening at 48 and 59 months postreconstruction affected two patients in the cemented INCS group, whereas no patients in the cementless INCS group experienced this complication (p=0.199) (**Figure 2**). Local recurrence affected one patient in the cemented INCS group who ultimately died of lung metastases at 22 months postreconstruction, whereas no patients in the cementless INCS group experienced local disease recurrence. None of the included patients experienced vascular incidents, nerve palsy, implant fractures, or periprosthetic infections (**Table 2**).

**Table 2:** Results for patients undergoing hip-preserving reconstruction with an intra-neck curved stem.

	N (%)			
Characteristic	Total (N=24)	HPR with cemented INCS (N=11)	HPR with cementless INCS (N=13)	*p*
Follow-up, mean (SD) (mo) Complications Aseptic loosening Yes No Periprosthetic infection Yes No Periprosthetic fracture Yes No Implant breakage Yes No Local recurrence Yes No Secondary procedure Yes No Survival status Dead Alive Pain assessment Severe Mild None MSTS score Gait-limp Yes No Need for assist device Yes No	56.0 (22.9) 2 (8.3) 22 (91.7) 0 24 (100) 0 24 (100) 0 24 (100) 1 (4.2) 23 (95.8) 2 (8.3) 22 (91.7) 4 (16.7) 20 (83.3) 0 3 (12.5) 21 (87.5) 25.6 (1.8) 0 24 (100) 0 24 (100)	63.5 (29.1) 2 (18.1) 9 (81.9) 0 11 (100) 0 11 (100) 0 11 (100) 1 (9.1) 10 (90.9) 2 (18.1) 9 (81.9) 3 (27.3) 8 (72.7) 0 2 (18.2) 9 (81.8) 24.9 (2.4) 0 11 (100) 0 11 (100)	49.7 (14.3) 0 (0) 13 (100) 0 13 (100) 0 13 (100) 0 13 (100) 0 13 (100) 0 (0) 13 (100) 1 (7.7) 12 (92.3) 0 1 (7.7) 12 (92.3) 26.0 (1.1) 0 13 (100) 0 13 (100)	0.175 0.199 — — — 0.458 0.199 0.300 0.439 0.168 — —

HPR, hip-preserving reconstruction; INCS, intra-neck curved stem; SD, standard deviation; MSTS, Musculoskeletal Tumor Society.

## Discussion

Performing HPR following MFMT resection remains a challenging procedure for musculoskeletal oncologists. In addition to the cemented and cementless fixation strategies used for distal femoral endoprostheses discussed herein, other treatment strategies have included the use of extracortical plates, cross-pin fixation, and compliant compression fixation. Compressive osseointegration fixation allows for the creation of a stable high-pressure interface between the bone and the implant, thereby potentially protecting against stress shielding (24, 25). This approach, however, is contraindicated in cases where the cortical thickness at the bone-implant surface is <2.5 mm (26). In children or patients who have undergone prior reconstructive procedures, the residual cortical thickness may be insufficient for this compression-based approach (27). Compression fixation has also been linked to various types of failure in cases of pathologic skeletal or extraskeletal conditions in which patients exhibit osteopenia and altered bone metabolism. These can include Type I failures defined by a combination of bone and interface failure, Type IIA failures defined by fracture proximal to the anchor plug, and Type IIB failures defined by fractures between the anchor plug and spindle (19). Chemotherapy can also reduce cortical hypertrophy rates at the bone-implant interface, leading to a reported downward trend in prosthetic survivorship (28). The complication rates associated with the use of stems with cross-fixation pins have been reported to be relatively low (29). However, this approach is associated with a need for more time to plan the procedure and manufacture the necessary customized systems, potentially making this approach infeasible for individuals undergoing neoadjuvant chemotherapy due to time constraints (27). Short-stemmed endoprostheses are theoretically susceptible to higher rates of aseptic loosening and implant failure (30), leading some to employ extra-cortical plants as a means of minimizing the risk of aseptic loosening through supplemental fixation. This can increase resistance to bending and rotational forces, particularly when additional screw fixation is performed. Standard side plate addition, together with a hydroxyapatite coating, is available from Stanmore (Stanmore Implants Worldwide Ltd, Elstree, UK), but its impact on overall survival remains to be assessed (19).

**Figure 3:**
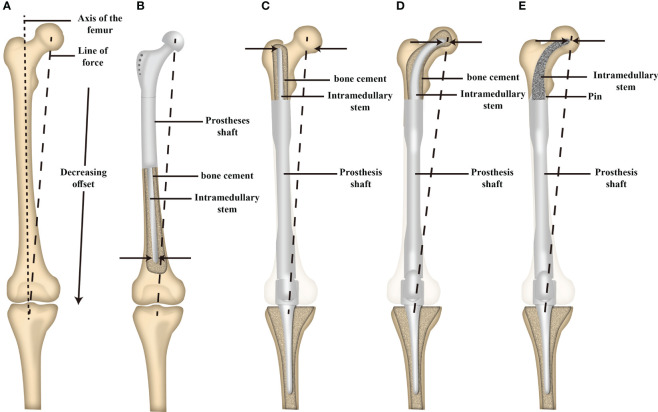
Schematic illustration of the offset distance between the line of force and the long axis of the femur and the offset distance of the tip of intramedullary stem between a proximal and distal femoral replacement. **(A)** The offset distance between the line of force and the long axis of the femur. **(B)** The offset distance of the tip of the intramedullary stem of proximal femoral replacement. **(C)** The offset distance of the tip of the intramedullary straight stem of distal femoral replacement. **(D)** The offset distance of the tip of the cemented INCS of distal femoral replacement. **(E)** The offset distance of the tip of the cementless INCS of distal femoral replacement. (INCS: intra-neck curved stem; Adapted from ref. (28) with permission).

The Compress ^®^ implant, cross-pin fixation, and stems with extracortical plates have been employed in MFMT patients undergoing HPR. The Compress ^®^ implant exhibited an 80% 10-year survival rate in a retrospective analysis of 82 patients performed by Healy et al. (26). Eckardt et al. further analyzed a cohort of 56 patients that underwent cross-pin endoprosthesis fixation, observing satisfactory durability outcomes at 10- and 15-year follow-up time points with 77% mechanical failure-free survivorship for distal femur implants at both time points (29, 31). A 70% 10-year survival rate has also been reported for stems with extra-cortical plates by Stevenson et al. (21) when evaluating reconstruction performed at multiple anatomic sites with customized implants with side plates to overcome short segment fixation and reconstruction challenges following extensive resection. In the present study, 2/24 MFMT patients undergoing HPR exhibited aseptic loosening at a mean follow-up time point of 56 months postreconstruction, with this aseptic loosening rate being consistent with those for the above-mentioned methods. There are several potential explanations for this outcome. For one, the endoprostheses used for HPR in these patients were designed to fit well with the local proximal femoral anatomy, and accurate INCS positioning was confirmed postoperatively. Second, relative to straight stems, the tip of the INCS is associated with a smaller offset distance and a smaller bending moment, potentially contributing to lower aseptic loosening rates (**Figure 3**) (32). In addition, INCS makes the force distribution of the cancellous bone more even (**Supplementary Figure 1**). Third, providing lasting fixation between bone and endoprostheses can be challenging for cemented prostheses, owing to a lack of sufficient residual proximal femoral length. When insufficient residual femoral length is available, a straight intramedullary stem will often exhibit a proximal endpoint within the trochanteric region, which contains inadequate cancellous bone and exhibits a large offset (30). When inadequate cancellous bone is available, this can impact bone cement interdigitation, with the resultant distribution and thickness of this bone cement impacting intramedullary endoprosthesis stability (33).

For distal femoral endoprosthetic reconstruction, Unwin and colleagues (34) reported 5% aseptic loosening, with the implant survivorship of 65% at 10 years and 53% at 20 years in 218 cases using cemented implants. The same group demonstrated 67.4% survival of aseptic loosening in custom, cemented distal femoral replacement at 10 years follow-up (30). Compared to cemented fixation, a low rate of aseptic loosening in cementless stems was reported by Griffin et al. (20), with femoral loosening identified in two cases (2.7%), between 1989 and 2000. Although there were significant design improvements on subsequent cementless systems, some studies have failed to demonstrate a significant difference in aseptic loosening rates between cemented and cementless stems in distal femoral replacement (35). In a retrospective comparative study of 232 patients who underwent lower limb tumor resection and reconstruction, Pala and colleagues (1) reported higher aseptic loosening in cemented stems (4.3%) than cementless stems (1.8%) at an average follow-up of just over 2 years. However, no statistically significant difference was found in that study. In the present study, the mean length of residual proximal femur in cemented INCS and cementless INCS was 90.6 and 69.7 mm, respectively (p<0.001). Although the residual short proximal femoral length was associated with a high risk of aseptic loosening and we did note gross differences in the rate of aseptic loosening in the study groups, the rate of aseptic loosening did not reach statistical significance (p=0.199). This may be attributed to the reduced offset distance of the curved stems compared to the straight stems, the appropriate cement technique and press-fit fixation of the curved stem with optimum diameter, and the surgical experience in oncological operations in specialized institution. Lee et al. (36) proposed that a 2–5-mm mantle is appropriate for the penetration of cement to bone. Moreover, increasing cement thickness seems an effective way to reduce stress in the bone and cement. In our study, for the purpose of increasing the stability of the INCS, the thickness of the bone cement at the base of the stem was moderately increased to 3–4 mm. In general, for HPR after the resection of MFMTs, cemented fixation could offer immediate stability and unrestricted weight-bearing in early outcomes. However, some studies reported that the survivorship of the cemented stem dropped precipitously in the long-term outcomes (30, 34). Additionally, some studies reported that cementless stem is coated with hydroxyapatite or 3D-printed porous titanium, which can facilitate biological bone ingrowth at the bone–prosthesis interface (37–39). At present, the current literature does not support the superiority of cementless over cemented implants. Longer-term longitudinal studies, ideally prospective randomized or retrospective match controlled, are required to define the appropriate reconstruction technique after the resection of MFMTs.

Average respective MSTS scores in patients in the cemented and cementless INCS groups were 24.9 and 26.0, in line with scores reported previously (40–42). While incomplete lower extremity functional rehabilitation was achieved in these patients, they nonetheless achieved sufficient pain relief and a level of limb function necessary to permit effective self-care. Moreover, the rapid postoperative recovery and return to full weight-bearing for patients undergoing such reconstructive surgery were particularly beneficial. No patients included in the present study cohort reported any postoperative limitations to lower limb function in their daily life. There may be several potential explanations for this finding. For one, the preservation of the native hip joint can minimize the risk of articular surface degeneration, muscular damage, and surgical disruption that may arise when employing prosthetic joints or osteoarticular allografts (6), thereby maximizing the functional recovery of the lower extremities. Second, the excellent stability of these endoprostheses and the return to natural bodyweight transmission may have led to better functional recovery. Third, the employed rehabilitative programs supported early functional training, contributing to better lower extremity functional recovery. MSTS scores did not differ between the two analyzed patient groups (p=0.168). While the length of femoral resection was longer in the cemented INCS group as compared to the cementless INCS group, the remaining proximal femur length in the cementless group was shorter than that in the cemented group (p<0.001). The rehabilitative programs employed herein were conducive to earlier functional training in individuals who have undergone procedures using a cemented INCS, allowing for greater benefit to the recovery of lower extremity function. Owing to these factors, MSTS scores did not differ between groups. Distal femur or diaphysis reconstruction is often associated with postoperative periprosthetic fracture and periprosthetic infection (31, 43, 44). However, no patients in the included study have experienced either of these outcomes as of the most recent follow-up.

There are certain limitations to this article. For one, this was a single-center study of procedures performed by one surgeon. Moreover, this was a retrospective analysis with a small sample size and a relatively short follow-up, potentially resulting in uncommon complications having been overlooked. Further studies with larger sample sizes and longer follow-up period are required to confirm the findings. Moreover, the relationship between adjuvant treatments and patient outcomes was not explored given that HPR procedures in MFMT patients are relatively rare. However, this study is the first to have specifically explored outcomes associated with cemented and cementless INCS use in this patient population, and these results may thus be instructive, providing a foundation for further research.

## Conclusion

In summary, this study explored the preliminary outcomes associated with the use of cemented and cementless INCS approaches in MFMT patients undergoing HPR. These results support the overall safety and efficacy of this reconstructive procedure and suggest that low complication rates and good lower limb function can be achieved provided an individualized rehabilitative program is appropriately implemented. Moreover, the selection between cemented or cementless INCS in the clinic should be made based on patient-specific factors, with cementless INCS implementation being preferable in younger patients with good-quality bone, the potential for long-term survival, and the osteotomy site near the lesser trochanter, whereas cemented INCS use should be favored for individuals who are older, have a shorter life expectancy, or have poor bone quality.

## Data availability statement

The original contributions presented in the study are included in the article/**Supplementary Material**. Further inquiries can be directed to the corresponding authors.

## Ethics statement

This study was reviewed and approved by West China Hospital, Sichuan University. The patients/participants provided their written informed consent to participate in this study.

## Author contributions

QY and ML performed the research, analyzed the data and wrote the paper. LM, YL, YoZ, YW, and CZ collected and analyzed the data. YoZ and CT designed the research, supervised the research, and reviewed the original paper. All authors contributed to the article and approved the submitted version.

## Funding

The study was supported by the Science and Technology Research Program of Sichuan Province (2020YFS0036), 1.3.5 project for disciplines of excellence, West China Hospital, Sichuan University (No. ZYJC18036) and QingDao research institutes of SiChuan university, Research of biomedical materials and 3D printing related products(20GZ30301), Post-Doctor Research Project, West China Hospital, Sichuan University (20HXBH136).

## Acknowledgments

The authors would like to thank all the reviewers who participated in the review, as well as MJEditor (www.mjeditor.com) for providing English editing services during the preparation of this manuscript.

## Conflict of interest

The authors declare that the research was conducted in the absence of any commercial or financial relationships that could be construed as a potential conflict of interest.

## Publisher’s note

All claims expressed in this article are solely those of the authors and do not necessarily represent those of their affiliated organizations, or those of the publisher, the editors and the reviewers. Any product that may be evaluated in this article, or claim that may be made by its manufacturer, is not guaranteed or endorsed by the publisher.

## Supplementary material

Supplementary Figure 1
**(A)** Finite element analysis of cancellous bone stress distribution after straight stem reconstruction. a) Residual proximal femur; b) Residual proximal femur coronal plane. **(B)** Finite element analysis of cancellous bone stress distribution after curved stem reconstruction. a) Residual proximal femur; b) Residual proximal femur coronal plane.

## References

[1] Pala E Mavrogenis AF Angelini A Henderson ER Douglas Letson G Ruggieri P. Cemented versus cementless endoprostheses for lower limb salvage surgery. J BUON Off J Balkan Union Oncol (2013) 18(2):496–503. 23818368

[2] Sevelda F Schuh R Hofstaetter JG Schinhan M Windhager R Funovics PT. Total femur replacement after tumor resection: Limb salvage usually achieved but complications and failures are common. Clin Orthop Relat Res (2015) 473(6):2079–87. doi: 10.1007/s11999-015-4282-1 PMC4419011 25832007

[3] Liu T Zhang X Zhang Q Zhang X Guo X. Total femoral reconstruction with custom prosthesis for osteosarcoma. World J Surg Oncol (2016) 14:93. doi: 10.1186/s12957-016-0852-2 PMC4815190 27030028

[4] Florian Sevelda WW Panotopoulos J MSc AK Funovics PT. Reinhard windhager is total femur replacement a reliable treatment option for patients with metastatic carcinoma of the femur? Clin Orthop Relat Res (2018) 476(5):977–83. doi: 10.1007/s11999.0000000000000125 PMC5916617 29480890

[5] Kalra S Abudu A Murata H Grimer RJ Tillman RM Carter SR. Total femur replacement: primary procedure for treatment of malignant tumours of the femur. Eur J Surg Oncol (2010) 36(4):378–83. doi: 10.1016/j.ejso.2009.11.002 20230929

[6] Dipak B Ramkumar SPK Niveditta Ramkumar LBE Santiago Lozano-Calderon MCG Megan E Anderson. Oncological and functional outcomes in joint-sparing resections of the proximal femur for malignant primary bone tumors;. J Pediatr Orthop (2021) 41(8):e680–e85. doi: 10.1097/BPO.0000000000001878 34091557

[7] Li Y Yang Y Huang Z Shan H Xu H Niu X. Bone defect reconstruction with autologous bone inactivated with liquid nitrogen after resection of primary limb malignant tumors. Medicine (2020) 99(24):e20442. doi: 10.1097/MD.0000000000020442 32541466 PMC7302590

[8] Bus MP van de Sande MA Taminiau AH Dijkstra PD. Is there still a role for osteoarticular allograft reconstruction in musculoskeletal tumour surgery? a long-term follow-up study of 38 patients and systematic review of the literature. Bone Joint J (2017) 99-b(4):522–30. doi: 10.1302/0301-620X.99B4.BJJ-2016-0443.R2 28385943

[9] Errani C Ceruso M Donati DM Manfrini M. Microsurgical reconstruction with vascularized fibula and massive bone allograft for bone tumors. Eur J orthopaedic Surg traumatology orthopedie traumatologie (2019) 29(2):307–11. doi: 10.1007/s00590-018-2360-2 30519732

[10] Ayvaz M Göker B Leblebicioğlu G. Hip-preserving reconstruction of the proximal femur with a vascularized fibula autograft and liquid-nitrogen-treated tumor bearing bone. Jt Dis Relat Surg (2021) 32(3):792–7. doi: 10.52312/jdrs.2021.12 PMC8650678 34842116

[11] Bus MP Dijkstra PD van de Sande MA Taminiau AH Schreuder HW Jutte PC. Intercalary allograft reconstructions following resection of primary bone tumors: a nationwide multicenter study. J Bone Joint Surg Am (2014) 96(4):e26. doi: 10.2106/JBJS.M.00655 24553895

[12] Aponte-Tinao LA Ayerza MA Albergo JI Farfalli GL. Do massive allograft reconstructions for tumors of the femur and tibia survive 10 or more years after implantation? Clin Orthop Relat Res (2020) 478(3):517–24. doi: 10.1097/CORR.0000000000000806 PMC7145084 32168064

[13] Li J Wang Z Guo Z Chen GJ Fu J Pei GX. The use of allograft shell with intramedullary vascularized fibula graft for intercalary reconstruction after diaphyseal resection for lower extremity bony malignancy. J Surg Oncol (2010) 102(5):368–74. doi: 10.1002/jso.21620 20872944

[14] Houdek MT Wagner ER Stans AA Shin AY Bishop AT Sim FH. What is the outcome of allograft and intramedullary free fibula (Capanna technique) in pediatric and adolescent patients with bone tumors? Clin Orthop Relat Res (2016) 474(3):660–8. doi: 10.1007/s11999-015-4204-2 PMC4746162 25701001

[15] Campanacci DA Totti F Puccini S Beltrami G Scoccianti G Delcroix L. Intercalary reconstruction of femur after tumour resection: is a vascularized fibular autograft plus allograft a long-lasting solution? Bone Joint J (2018) 100-b(3):378–86. doi: 10.1302/0301-620X.100B3.BJJ-2017-0283.R2 29589494

[16] Rabitsch K Maurer-Ertl W Pirker-Fruhauf U Wibmer C Leithner A. Intercalary reconstructions with vascularised fibula and allograft after tumour resection in the lower limb. Sarcoma (2013) 2013:160295. doi: 10.1155/2013/160295 23766665 PMC3676952

[17] Frisoni T Cevolani L Giorgini A Dozza B Donati DM. Factors affecting outcome of massive intercalary bone allografts in the treatment of tumours of the femur. J Bone Joint Surg Br (2012) 94(6):836–41. doi: 10.1302/0301-620X.94B6.28680 22628602

[18] Bhangu AA Kramer MJ Grimer RJ O'Donnell RJ. Early distal femoral endoprosthetic survival: cemented stems versus the compress implant. Int Orthop (2006) 30(6):465–72. doi: 10.1007/s00264-006-0186-8 PMC3172732 16983554

[19] Christ AB Hornicek FJ Fabbri N. Distal femoral replacement - cemented or cementless? current concepts and review of the literature. J Clin Orthop Trauma (2021) 19:11–6. doi: 10.1016/j.jcot.2021.04.032 PMC8138588 34040980

[20] Griffin AM Parsons JA Davis AM Bell RS Wunder JS. Uncemented tumor endoprostheses at the knee: root causes of failure. Clin Orthop Relat Res (2005) 438:71–9. doi: 10.1097/01.blo.0000180050.27961.8a 16131872

[21] Stevenson JD Wigley C Burton H Ghexelayagh S Morris G Evans S. Minimising aseptic loosening in extreme bone resections: custom-made tumour endoprostheses with short medullary stems and extra-cortical plates. Bone Joint J (2017) 99-b(12):1689–95. doi: 10.1302/0301-620X.99B12.BJJ-2017-0213.R1 29212694

[22] Enneking WF. A system of staging musculoskeletal neoplasms. Instructional course lectures (1988) 37:3–10. 3047253

[23] Enneking WF Dunham W Gebhardt MC Malawar M Pritchard DJ. A system for the functional evaluation of reconstructive procedures after surgical treatment of tumors of the musculoskeletal system. Clin Orthop Relat Res. (1993) (286):241–6. doi: 10.1007/978-1-4471-5451-8_128 8425352

[24] Kramer MJ Tanner BJ Horvai AE O'Donnell RJ. Compressive osseointegration promotes viable bone at the endoprosthetic interface: retrieval study of compress implants. Int Orthop (2008) 32(5):567–71. doi: 10.1007/s00264-007-0392-z PMC2551719 17576554

[25] Kagan R Adams J Schulman C Laursen R Espana K Yoo J. What factors are associated with failure of compressive osseointegration fixation? Clin Orthop Relat Res (2017) 475(3):698–704. doi: 10.1007/s11999-016-4764-9 PMC5289163 26926774

[26] Healey JH Morris CD Athanasian EA Boland PJ. Compress knee arthroplasty has 80% 10-year survivorship and novel forms of bone failure. Clin Orthop Relat Res (2013) 471(3):774–83. doi: 10.1007/s11999-012-2635-6 PMC3563794 23054526

[27] Hindiskere S Staals E Donati DM Manfrini M. What is the survival of the telescope allograft technique to augment a short proximal femur segment in children after resection and distal femur endoprosthesis reconstruction for a bone sarcoma? Clin Orthop Relat Res (2021) 479(8):1780–90. doi: 10.1097/CORR.0000000000001686 PMC8277267 33635286

[28] Avedian RS Goldsby RE Kramer MJ O'Donnell RJ. Effect of chemotherapy on initial compressive osseointegration of tumor endoprostheses. Clin Orthop Relat Res (2007) 459:48–53. doi: 10.1097/BLO.0b013e3180514c66 17545758

[29] Cannon CP Eckardt JJ Kabo JM Ward WG Sr Kelly CM Wirganowicz PZ. Custom cross-pin fixation of 32 tumor endoprostheses stems. Clin Orthop Relat Res (2003) 417:285–92. doi: 10.1097/01.blo.0000096801.78689.9e 14646728

[30] Unwin PS Cannon SR Grimer RJ Kemp HB Sneath RS Walker PS. Aseptic loosening in cemented custom-made prosthetic replacements for bone tumours of the lower limb. J Bone Joint Surg Br (1996) 78(1):5–13. 8898118

[31] Bernthal NM Upfill-Brown A Burke ZDC Ishmael CR Hsiue P Hori K. Long-term follow-up of custom cross-pin fixation of 56 tumour endoprosthesis stems: a single-institution experience. Bone Joint J (2019) 101-b(6):724–31. doi: 10.1302/0301-620X.101B6.BJJ-2018-0993.R1 31154850

[32] Wyatt MC Kieser DC Kemp MA McHugh G Frampton CMA Hooper GJ. Does the femoral offset affect replacements? the results from a national joint registry. Hip Int (2019) 29(3):289–98. doi: 10.1177/1120700018780318 29873253

[33] Ebramzadeh E Sarmiento A McKellop HA Llinas A Gogan W. The cement mantle in total hip arthroplasty. analysis of long-term radiographic results. J Bone Joint Surg Am (1994) 76(1):77–87. doi: 10.2106/00004623-199401000-00010 8288668

[34] Unwin PS Cobb JP Walker PS. Distal femoral arthroplasty using custom-made prostheses. the first 218 cases. J arthroplasty (1993) 8(3):259–68. doi: 10.1016/S0883-5403(06)80087-2 8326306

[35] Capanna R Scoccianti G Frenos F Vilardi A Beltrami G Campanacci DA. What was the survival of megaprostheses in lower limb reconstructions after tumor resections? Clin Orthop Relat Res (2015) 473(3):820–30. doi: 10.1007/s11999-014-3736-1 PMC4317421 24964884

[36] Lee IY Skinner HB Keyak JH. Effects of variation of cement thickness on bone and cement stress at the tip of a femoral implant. Iowa Orthopaedic J (1993) 13:155–9. PMC2328994 7820736

[37] Van der Stok J van der Jagt OP Amin Yavari S De Haas MF Waarsing JH Jahr H. Selective laser melting-produced porous titanium scaffolds regenerate bone in critical size cortical bone defects. J Orthop Res (2013) 31(5):792–9. doi: 10.1002/jor.22293 23255164

[38] Lu M Wang J Xiao C Tang F Min L Zhou Y. Uncemented, curved, short endoprosthesis stem for distal femoral reconstruction: early follow-up outcomes. World J Surg Oncol (2018) 16(1):183. doi: 10.1186/s12957-018-1486-3 PMC6131732 30200979

[39] Zhao D Tang F Min L Lu M Wang J Zhang Y. Intercalary reconstruction of the "Ultra-critical sized bone defect" by 3D-printed porous prosthesis after resection of tibial malignant tumor. Cancer Manage Res (2020) 12:2503–12. doi: 10.2147/CMAR.S245949 PMC7152541 32308487

[40] Zheng K Yu XC Hu YC Shao ZW Xu M Wang BC. Outcome of segmental prosthesis reconstruction for diaphyseal bone tumors: a multi-center retrospective study. BMC Cancer (2019) 19(1):638. doi: 10.1186/s12885-019-5865-0 PMC6599373 31253134

[41] Calvert GT Cummings JE Bowles AJ Jones KB Wurtz LD Randall RL. A dual-center review of compressive osseointegration for fixation of massive endoprosthetics: 2- to 9-year followup. Clin Orthop Relat Res (2014) 472(3):822–9. doi: 10.1007/s11999-013-2885-y PMC3916600 23467985

[42] Dieckmann R Henrichs MP Gosheger G Holl S Hardes J Streitburger A. Short-stem reconstruction for megaendoprostheses in case of an ultrashort proximal femur. BMC Musculoskelet Disord (2014) 15:190. doi: 10.1186/1471-2474-15-190 24885859 PMC4067112

[43] Choi HS Nho JH Kim CH Kwon SW Park JS Suh YS. Revision arthroplasty using a MUTARS® prosthesis in comminuted periprosthetic fracture of the distal femur. Yonsei Med J (2016) 57(6):1517–22. doi: 10.3349/ymj.2016.57.6.1517 PMC5011288 27593884

[44] Bus MP van de Sande MA Fiocco M Schaap GR Bramer JA Dijkstra PD. What are the long-term results of MUTARS((R)) modular endoprostheses for reconstruction of tumor resection of the distal femur and proximal tibia? Clin Orthop Relat Res (2017) 475(3):708–18. doi: 10.1007/s11999-015-4644-8 PMC5289150 26649558

