# Supplementary figures and images for: A comparison of cemented and cementless intra-neck curved stem use during hip-preserving reconstruction following massive femoral malignant tumor removal
Source: Front Oncol. 2022 Sep 5;12:933057. doi: 10.3389/fonc.2022.933057 (PMC9483172; doi:10.3389/fonc.2022.933057)

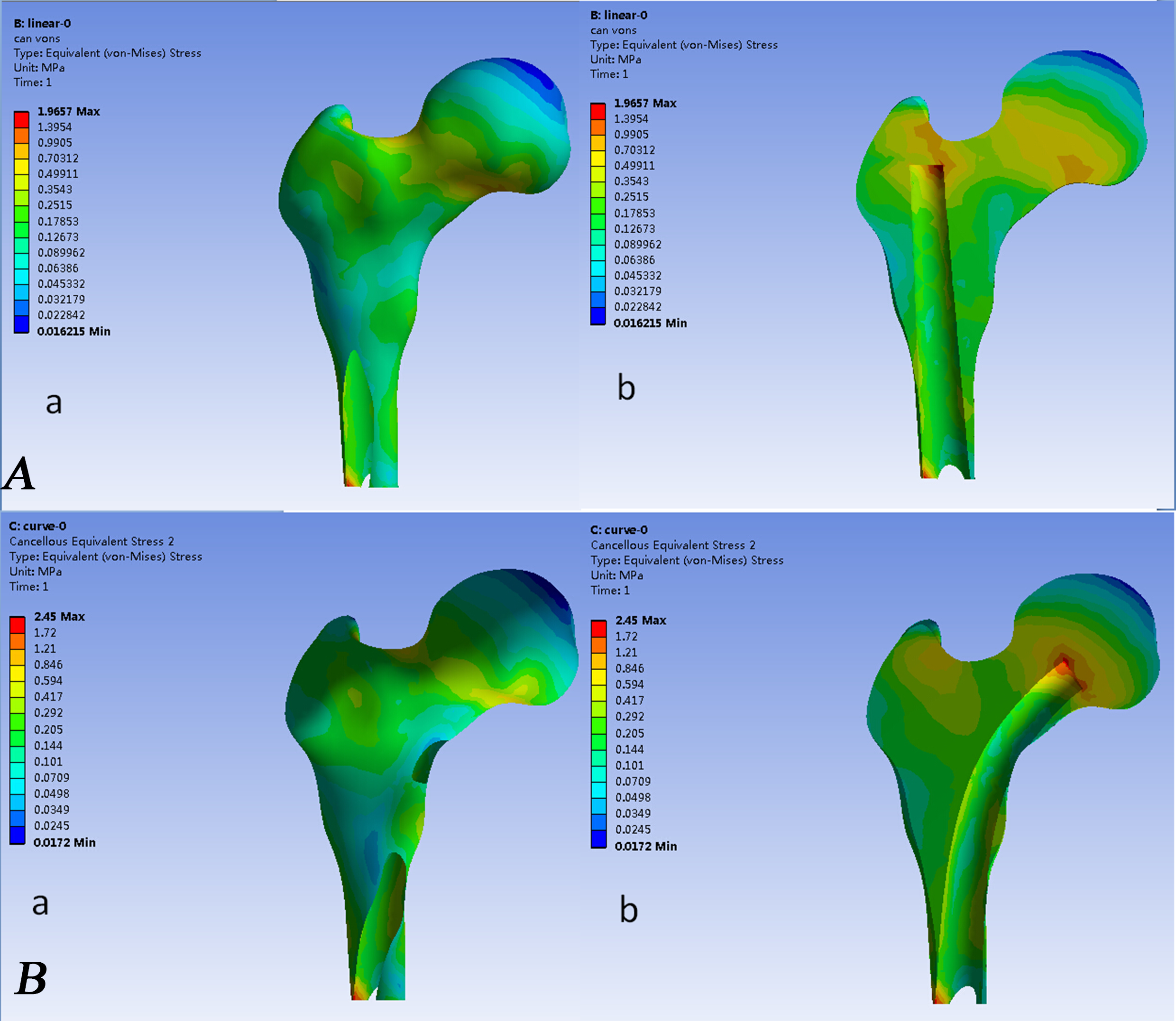

Supplement: Supplementary Figure 1 — (A) Finite element analysis of cancellous bone stress distribution after straight stem reconstruction. a) Residual proximal femur; b) Residual proximal femur coronal plane. (B) Finite element analysis of cancellous bone stress distribution after curved stem reconstruction. a) Residual proximal femur; b) Residual proximal femur coronal plane. [file Image_1.tif]
